# Evolution of the eukaryotic protein kinases as dynamic molecular switches

**DOI:** 10.1098/rstb.2012.0054

**Published:** 2012-09-19

**Authors:** Susan S. Taylor, Malik M. Keshwani, Jon M. Steichen, Alexandr P. Kornev

**Affiliations:** 1 Department of Chemistry and Biochemistry, University of California San Diego, La Jolla, CA 92093, USA; 2 Department of Pharmacology, University of California San Diego, La Jolla, CA 92093, USA; 3 Howard Hughes Medical Institute, University of California San Diego, La Jolla, CA 92093, USA

**Keywords:** phosphorylation, protein kinases, evolution

## Abstract

Protein kinases have evolved in eukaryotes to be highly dynamic molecular switches that regulate a plethora of biological processes. Two motifs, a dynamic activation segment and a GHI helical subdomain, distinguish the eukaryotic protein kinases (EPKs) from the more primitive eukaryotic-like kinases. The EPKs are themselves highly regulated, typically by phosphorylation, and this allows them to be rapidly turned on and off. The EPKs have a novel hydrophobic architecture that is typically regulated by the dynamic assembly of two hydrophobic spines that is usually mediated by the phosphorylation of an activation loop phosphate. Cyclic AMP-dependent protein kinase (protein kinase A (PKA)) is used as a prototype to exemplify these features of the PKA superfamily. Specificity in PKA signalling is achieved in large part by packaging the enzyme as inactive tetrameric holoenzymes with regulatory subunits that then are localized to macromolecular complexes in close proximity to dedicated substrates by targeting scaffold proteins. In this way, the cell creates discrete foci that most likely represent the physiological environment for cyclic AMP-mediated signalling.

## 1. Introduction

The concept of protein phosphorylation as a mechanism for regulation began with the pioneering studies of Krebs and Fischer in the middle of the last century [1,2]. These studies, which demonstrated that glycogen phosphorylase was activated by the reversible addition of a single phosphate, laid the foundation for the family of eukaryotic protein kinases (EPKs). We now recognize that this family, termed as the ‘kinome’, represents one of the largest gene families encoded by most eukaryotic genomes. In humans, there are over 500 EPKs [3], and they regulate most of the biological processes that take place in the cell. How did these unique proteins evolve, and how are they different from the metabolic enzymes that we have studied in such depth? We discuss here the essential features that define the EPKs and distinguish them from the earlier and simpler eukaryotic-like kinases (ELKs). Because we now have a significant ‘structural’ kinome available that contains over 150 protein kinases [4], we can also elucidate the general unique features of the EPK family as well as features that are associated with each subfamily. Using cyclic AMP (cAMP)-dependent protein kinase (protein kinase A (PKA)) as a model, we will also define how our understanding of the structure and regulation of one protein kinase has served as a prototype for the overall family.

cAMP was discovered by Sutherland as a hormone second messenger about the same time that Krebs and Fischer discovered protein phosphorylation as a regulatory mechanism [5,6]. PKA was discovered about a decade later as the kinase that phosphorylated and activated phosphorylase kinase [7]. The discovery of the PKA regulatory subunits as the primary receptor for cAMP provided the mechanism for having PKA under the control of cAMP [8–11]. Each PKA R-subunit contains two contiguous cyclic nucleotide-binding (CNB) domains, and these CNB domains are widespread in the prokaryotic world [12]. They appear to be an ancient motif that has co-evolved with cAMP as a mechanism for translating the stress-induced cAMP second messenger into a biological response. There are examples of CNB domains being coupled to protein kinase domains in prokaryotic gene sequences, so these motifs have been functionally coupled for a very long time, but none of these have been studied at the protein level. The closest homologue to PKA is cyclic GMP (cGMP)-dependent protein kinase (protein kinase G (PKG)), but in this case the kinase domain is fused to the CNB domains [13,14]. Both cAMP and cGMP domains are first found functionally linked to an EPK early in the evolution of eukaryotes. They are found, for example, in all fungi [15] and even in single cell symbiotic pathogens such as trypanosomes [16] and plasmodia [17].

cAMP is generated in response to the activation of G protein-coupled receptors (GPCRs) through the activation of Gαs, the activating G-protein subunit that stimulates adenylate cyclase [18]. Over 30 GPCRs couple to Gαs, and there are four functionally non-redundant PKA R-subunits. The inactive PKA holoenzymes are localized to specific sites in the cell by scaffold proteins, the best known being the A kinase anchoring proteins (AKAPs) [19]. These proteins constitute a diverse PKA signalling system that exists in every mammalian cell and provides a mechanism for achieving exquisite specificity. The system is defined by what GPCRs, PKAs and AKAPs are expressed in each cell. These proteins are then assembled into discrete localized ‘foci’ of PKA signalling. Understanding how these macromolecular complexes are assembled and regulated is our challenge for the future. Elucidating structures of the individual proteins is only the first step. Now that we have well-defined kinases, we must understand how each works in the context of the whole cell. To achieve such an integrated understanding of signalling requires many disciplines that include computational strategies to link the proteins into well-defined pathways.

## 2. Kinase core

**Figure 1.**
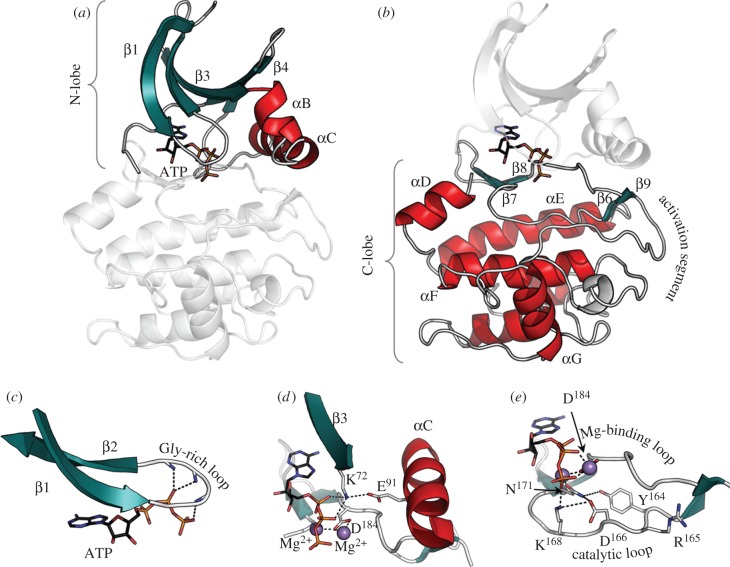
Conserved core of the eukaryotic protein kinases. The bottom panels (*c*–*e*) highlight functional motifs in the N-lobe (*a*) and the C-lobe (*b*) using PKA as a prototype for the EPK family. Helices are shown in red; β-strands in teal. (*a*) The N-lobe contains five β-strands and a large αC-helix. (*b*) The C-lobe is mostly helical with a large activation segment. A four-stranded β-sheet rests on the helical core and forms one surface of the active site cleft. ATP is bound in the cleft between the two lobes. (*c*) The phosphates of ATP are positioned by a conserved glycine-rich loop between the β1- and β2-strands. (*d*) Conserved residues Lys72 from the β3-strand, Glu91 from the αC-helix, and Asp164 from the DFG motif in the activation segment where Mg^2+^ ions are show as purple balls. (*e*) The catalytic loop also contains a set of catalytically important residues: Asp166, Lys168, Asn171.

The EPK superfamily is defined by a conserved kinase core. This core consists of a bi-lobal protein of approximately 250 residues that contains most of the essential machinery for catalysis and for scaffolding, the two functions that are essential for downstream signalling (figure 1). Scattered throughout the core are specific conserved sequence motifs that were classified early on into 12 subdomains by Hanks & Hunter [20]. Once the crystal structure of PKA was solved, these motifs could be defined in terms of their putative function [21,22]. Each lobe is made up of helical and beta subdomains, and the active site cleft is formed by the two lobes converging to form a deep cleft where the adenine ring of adenosine triphosphate (ATP) is bound. In this configuration, which is quite distinct from the Walker motif that is associated with most other ATP-binding proteins [23], the γ-phosphate is positioned at the outer edge of the cleft where phosphoryl transfer takes place. Catalysis is mediated by opening and closing of the active site cleft allowing for transfer of the phosphate and then release of the nucleotide.

The N-terminal lobe (N-lobe) consists of a five-stranded anti-parallel beta sheet that is an essential part of the ATP-binding mechanism. Between β3 and β4 is the single conserved helix, the αC-helix. β-Strands 1 and 2 are joined by a glycine-rich loop, and this loop packs on top of the ATP with a backbone amide at the tip of the loop anchoring the γ-phosphate and positioning it for phosphoryl transfer. β-Strand 3 contains an essential Lys (Lys72 in PKA) that couples to a conserved Glu (Glu91 in PKA) in the αC-helix when the kinase is in an active conformation. The conserved helical subdomain in the N-lobe of PKA and other AGC kinases contains a short αB-helix and a long αC-helix. The αB-helix is not conserved in all kinases, but the αC-helix is an essential conserved feature of every protein kinase. When a kinase is in an active conformation, the N-terminus of the αC-helix typically interacts with the activation loop phosphate (see later text), while the C-terminus is part of the hinge at the base of the active site cleft.

In contrast to the N-lobe, the C-terminal lobe (C-lobe) is mostly helical and quite stable conformationally. The αE-, αF- and αH-helices, based on hydrogen–deuterium exchange, for example, are very stable and do not exchange much with solvent even after several days [24]. Resting on the helical core is a four-stranded β sheet that contains most of the remaining catalytic residues (figure 1). This β sheet forms the bottom surface of the active site cleft as seen in the classic view of a protein kinase. Between β-strands 6 and 7 lies the catalytic loop (H/YRDXKXXN) (Asp164 in PKA is the catalytic base) that corresponds to subdomain VIb. This segment contains many of the key residues that direct the γ-phosphate of ATP to the protein substrate. Between β-strands 8 and 9, referred to as the magnesium positioning loop, is the Asp-Phe-Gly (DFG) motif where a conserved aspartate (Asp184 in PKA) binds to the catalytic magnesium ion. Although substrates can be tethered to either the N-lobe or the C-lobe or through a flanking domain or linker in a way that positions the P-site to serve as the acceptor for the γ-phosphate, most are tethered to the C-lobe. This peptide containing the phosphorylation site (Ser, Thr or Tyr) lies along the outer surface of the C-lobe near the active site cleft.

## 3. The n- and c-lobes are connected by hydrophobic spines

**Figure 2.**
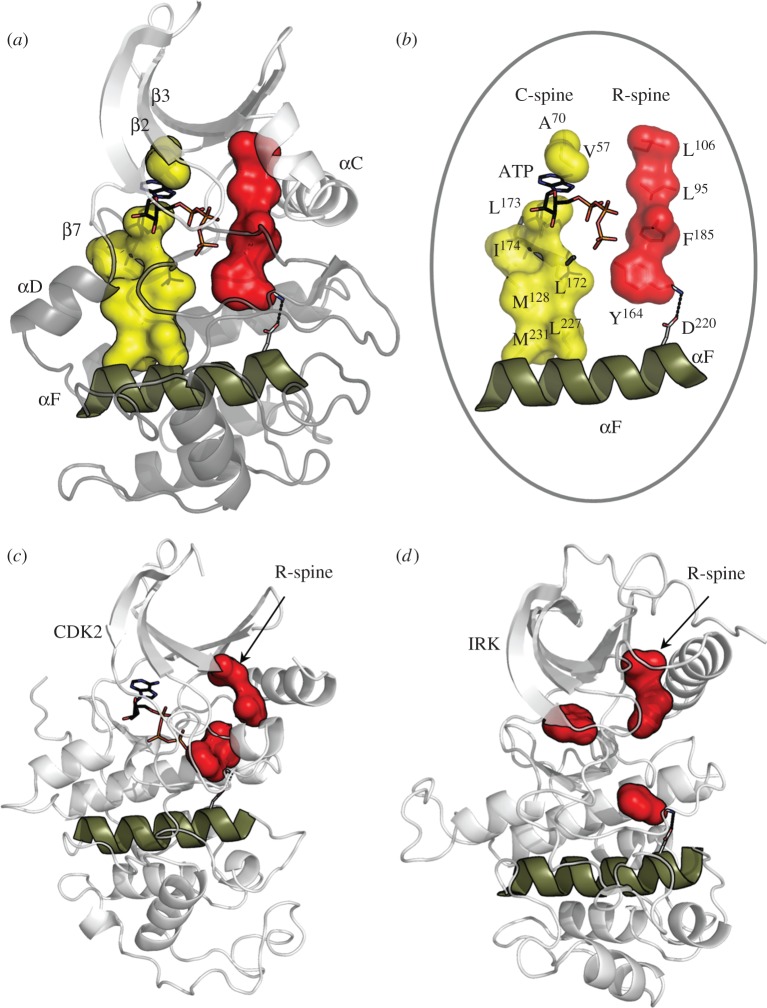
Hydrophobic spines define the internal architecture of the EPKs. (*a*) Two hydrophobic spines span the two lobes of the kinase core and provide a firm but flexible connection between the N- and C-lobes. (*b*) The regulatory spine (R-spine) contains four residues from different kinase subdomains and is anchored to the αF-helix by conserved Asp220. The catalytic spine (C-spine) is completed by ATP. (*c*,*d*) In the inactive state, the R-spine is typically disassembled. Disassembly of the R-spine can be achieved in different ways: by movement of the αC-helix like in cyclin-dependent kinase 2 (CDK2) (*c*) or by movement of the activation segment like in insulin receptor kinase (IRK) (*d*).

Solving the structure of PKA allowed us to see the conserved sequence motifs of the EPK family in three-dimensions and gave functional significance to each motif [21,22]. Having many protein kinase structures available allows us to search for additional conserved motifs. While the original well-defined sequence motifs were mostly hydrophilic and associated directly with ATP binding or catalysis, the analysis of many protein kinase structures provided insights into how hydrophobic residues contribute to the overall kinase assembly and architecture. A rigorous comparison of many protein kinase structures, both active and inactive, revealed that the core is built around a stable yet dynamic hydrophobic core that is made up of three essential elements—a single hydrophobic helix that spans the large lobe (αF-helix) and two hydrophobic spines that are each made up of non-contiguous residues from both lobes (figure 2). These spines are composed of nonlinear residues and would never be recognized as conserved spatial motifs based on sequence comparisons alone. The spines were first recognized when active and inactive kinases were compared using a method referred to as local spatial pattern (LSP) alignment [25]. This method allows one to rapidly compare any two related structures and to identify spatially conserved residues. What became clear in the comparison of active and inactive kinases is that every active kinase had a contiguous hydrophobic spine that is made up of two residues from the N-lobe and two from the C-lobe. Because this spine is broken in the inactive kinases, it was referred to as a ‘regulatory’ spine or R-spine. Typically, the R-spine is assembled as a consequence of phosphorylation of the activation loop that joins β-strand 9 to the αF-helix. Most often this causes the DFG motif that lies between β-strands 8 and 9 to ‘flip’ so that the phenylalanine (Phe185 in PKA) is positioned to complete the spine, and leaving the Asp positioned to interact with one of the ATP bound magnesium ions. In general, the ‘DFG-in’ conformation refers to the completed R-spine, whereas the ‘DFG-out’ conformation corresponds to a broken R-spine. There are, however, DFG-in conformations that still are inactive because the spine is broken in a different way, emphasizing that one needs to evaluate the overall spine configuration before classifying a particular structure as ‘active’ or ‘inactive’. The four non-contiguous residues that are aligned in every active kinase are the His from the His-Arg-Asp (HRD) motif in the catalytic loop that bridges β-strands 6 and 7. In PKA and some of the other AGC kinases, this His is replaced with a Tyr (Tyr164), but in each case this hydrophobic residue is packed against the Phe from the DFG motif. In the N-lobe of PKA, the two hydrophobic R-spine residues are the leucine Leu95 in the αC-helix and Leu106 in β-strand 5. These two residues are not only linked to the C-lobe through the R-spine but also serve to anchor the beta and helical subdomains in the N-lobe together [4].

The LSP method was then used to compare all protein kinases, and this revealed another hydrophobic ‘spine’ that runs parallel to the regulatory (R) spine. Unlike the R-spine, this second spine is completed not by the dynamic insertion of a hydrophobic amino acid side chain but rather by the adenine ring of ATP. This spine is therefore referred to as the ‘catalytic’ spine or C-spine. ATP binding thus positions the two lobes so that the catalytic residues are aligned optimally for catalysis. Both spines are anchored to the αF-helix. The catalytic loop is also firmly anchored through hydrophobic residues to the αF-helix. This hydrophobic core is a conserved feature of all EPKs and is quite distinct from the hydrophobic core of most globular proteins. Assembly of the R-spine in such a dynamic way is a fundamental feature of the ‘switch’ mechanism.

Outliers of the EPK family are the phosphoinositide 3-kinases (PI-3 kinases) that share much of the catalytic machinery that is found in the EPKs and ELKs. However, the GHI subdomain is different as is the activation loop. These PI-3 kinases, nevertheless, do have the remnants of the two hydrophobic R- and C-spines [26].

## 4. Evolution of the eukaryotic protein kinases

**Figure 3.**
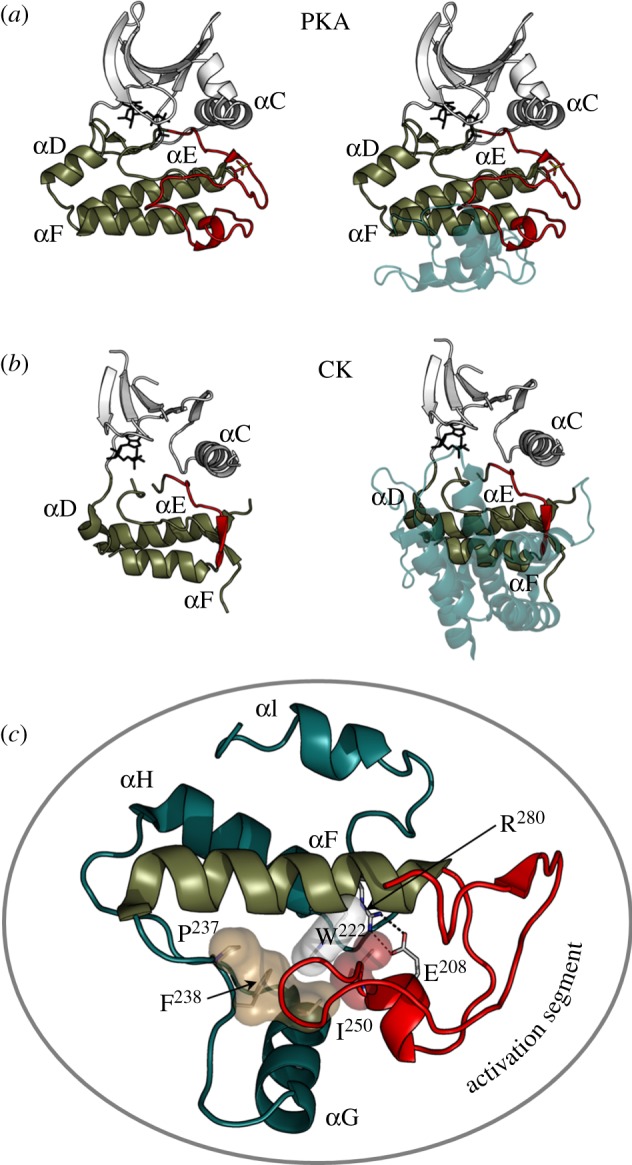
Activation segment and the helical GHI subdomain distinguish EPKs from ELKs. The activation segment (shown in red) that joins the DFG motif to the αF-helix is shown on the left side of the two top panels while the helical GHI subdomains that follow the αF-helix are shown on the right and indicated in teal. (*a*) PKA, a prototype for EPKs, is compared with a eukaryote-like kinase: (*b*) choline kinase. (*c*) The extended activation segment (red), which is typically regulated by phosphorylation, is a unique feature of the EPKs. The GHI helical subdomain is also conserved in EPKs and functions as a docking site and, most likely, as an allosteric link to the active site in EPKs. While the ELKs also have helical subdomains following the F-helix, these helical regions are not conserved with respect to each other or to the EPKs (shown in teal). The GHI subdomain and the activation segment are bound to each other and to the αF-helix via a set of conserved hydrophobic interactions (shown as transparent surfaces) and a buried salt bridge Glu208–Arg280.

The era of genome sciences has allowed us to delve deeply into the evolutionary history of proteins. From such genome-wide screens, we discover that EPKs evolved from much simpler ELKs that are abundant in prokaryotes [27]. Many of the ELKs, such as choline kinase [28] and amino glycoside kinase [29], are metabolic enzymes that act on small molecules. These ELKs also have a bi-lobal structure (figure 3). The N-lobe, in particular, that positions ATP for catalysis is very similar, including the conserved ion pair between Lys72 and Glu91. A portion of the C-lobe, through the αF-helix, is also conserved. The EPKs are distinguished from the ELKs, however, by two unique co-evolved elements—the activation segment that links β-strand 9 to the αF-helix and the GHI helical subdomain (figure 3). While the activation segment in ELKs is short and not regulated, in EPKs it is highly dynamic and is typically assembled in its active conformation by the addition of a phosphate that is mediated by either *cis-* or *trans*-autophosphorylation or by the action of a heterologous activating kinase (*trans*). This activation by phosphorylation is a characteristic feature of most EPKs, and in some cases, such as ribosomal S6 kinase (RSK), there are 4–5 kinases that contribute to the activation of a single kinase [30]. A few kinases do not require phosphorylation for their activation. This complex regulation by phosphorylation emphasizes that the EPKs have evolved to be highly dynamic ‘switches’ that initiate a downstream signalling event.

The GHI helical subdomain, which has co-evolved with the complex and dynamic activation loop in EPKs, is also missing in the ELKs. This subdomain provides docking sites for tethering protein substrates and also provides additional regulatory/allosteric control. Both the GHI helical subdomain and the activation loop are anchored firmly to the hydrophobic αF-helix and are linked to each other by a highly conserved and buried ion pair that is a hallmark signature motif of the EPKs. This ion pair consists of a conserved arginine (Arg 280 in PKA) that lies between the αH- and αI-helices and a conserved glutamate (Glu208 in PKA), which is at the end of the activation loop and part of the Ala-Pro-Glu (APE) motif. The segment that lies between the APE motif and the αF-helix is also a structurally conserved element; it serves as a hydrophobic anchor that locks the activation loop onto the αF-helix [4]. Included in this region is also a conserved tyrosine (Tyr215 in PKA) that reaches back to the phosphorylation site in the activation loop. The Arg–Glu ion pair provides a sensitive allosteric link to the active site [31]. Mutation of either residue, Glu208 or Arg280 in PKA, has a profound effect on catalytic activity through an increase in *K*_m_ (ATP) and a decrease in *k*_cat_. The αH-helix is an allosteric regulatory site in the yeast homologue of PKA, tpk1, and provides a pathway for substrates to communicate with the active site [32]. This Glu208–Arg280 ion pair thus serves as a central hub of connectivity between these two structurally conserved elements and is a defining feature that distinguishes the EPKs from the ELKs [31].

## 5. Linkers and tails

**Figure 4.**
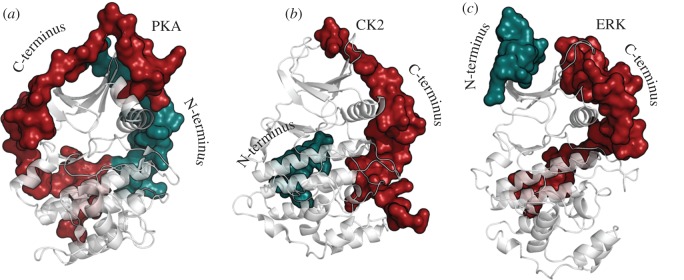
Tails and linkers. Flanking regions wrap around the conserved kinase core in different ways for the various kinases but typically, the kinase core alone is not sufficient for optimal activity. These tails provide stability and allosteric mechanisms for regulation. Three EPK examples are shown: (*a*) protein kinase A (PKA), (*b*) casein kinase II (CK2) and (*c*) ERK2. Each kinase core is displayed as a ribbon. The N-terminal tails are shown as a teal surface while the C-terminal tails are in red.

Although the core that defines the protein kinase superfamily contains most of the essential catalytic machinery, it is surprisingly not usually sufficient to mediate optimal catalysis on its own. Instead, it is assembled into a fully active state by interactions with flanking regions or domains that are anchored to the core (figure 4). In the case of PKA there is a short N-tail (39 residues) and C-tail (50 residues) that wrap around both lobes of the core. Without these tails, the kinase is neither stable nor active. Mitogen-activated kinase is similar in that it has N- and C-tails that wrap around the core in different ways but nevertheless achieve the same function of stabilizing the active kinase and contributing to catalysis [33]. The non-receptor tyrosine kinases, such as Src, typically have two N-terminal domains, an SH2 and an SH3 domain, and these interact with the core to keep it off or on [34]. Specifically, the SH2 and SH3 domains interact with the catalytic kinase core domain to maintain an inactive conformation as exemplified by Src and Abl, whereas the SH2 domain can interact with the N-lobe to promote activity when the kinase inhibition is released as seen in Abl and Fes [35]. The receptor tyrosine kinases are the most challenging because they contain a large extracellular domain that binds typically to a growth factor. Binding of the growth factor to the extracellular domains promotes dimerization and also releases the inhibition of the cytoplasmic kinase domain. In addition to a single transmembrane (TM) helix, there is a linker region that joins the TM helix to the kinase core and also a C-terminal tail. The linker and the C-tail are filled with various phosphorylation sites that constitute a highly sophisticated regulatory mechanism. Transitioning of these linkers between ordered and disordered states is a critical part of the signalling mechanism but extremely difficult to trap in a crystal lattice.

**Figure 5.**
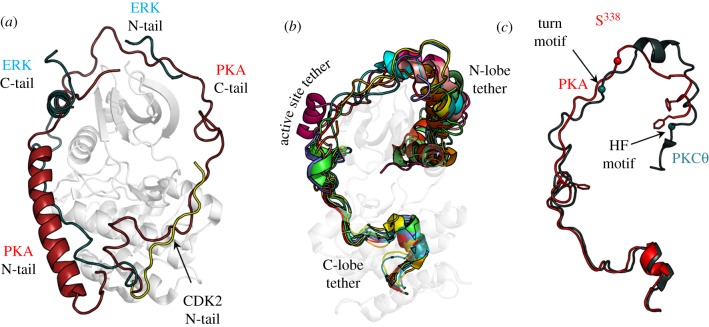
The activation loops and the C-tails are regulated by phosphorylation. (*a*) Shows how the tails from the three different kinases shown in figure 4 wrap around the core in different ways but fill the same space. (*b*) The C-tails are a conserved feature of the AGC subfamily of EPKs. (*c*) The C-tail of most AGC kinases, exemplified here by PKC ζ (teal), are assembled into an active conformation by phosphorylation at a turn motif and at the hydrophobic motif near the C-terminus. The C-tail of PKA (red) ends with the HF motif and lacks the final phosphorylation site.

The highly sophisticated ways in which the tails can contribute to both regulation and catalysis can perhaps best be appreciated by looking more closely at the PKA catalytic subunit and the AGC subfamily (figure 5). PKA is flanked at its N-terminus by an N-terminal myristoyl moiety that is followed by an amphipathic helix. The hydrophobic surface of the helix is anchored to the large lobe of the kinase core and to a critical hinge socket that lies at the base of the active site cleft [36]. The hydrophilic surface of the helix provides a docking site for another PKA interacting protein called A kinase interacting protein 1 (AKIP1). AKIP1 is involved in trafficking the catalytic subunit to the nucleus [37]. This N-terminal helix is not conserved in the AGC subfamily although other motifs and domains play a similar role in docking to this surface of the core.

The C-tail, however, is highly conserved in all members of the AGC subfamily and is also highly regulated by phosphorylation, both *cis* and *trans* [38]. The C-tail of PKA, and indeed of all AGC protein kinases, can be divided into three functionally distinct segments. The first segment (residues 301–318), referred to as the C-lobe tether, is bound firmly to the large C-lobe of the kinase core. Several conserved motifs are embedded within this segment, including a PXXP motif that was shown in PKCα to bind to Hsp70 [39]. This is followed by a highly dynamic segment, the active site tether (AST). This segment contains an FDX(X)Y/F motif that is an integral part of the ATP-binding site. The first Phe is part of the adenine-binding pocket, and mutation of this Phe results in significant loss of activity [40]. The second aromatic residue (Phe or Tyr) lies on top of the ribose ring when the enzyme is in a closed conformation. Mutation of Tyr330 also leads to loss of activity in PKA [41]. In the absence of bound nucleotide, the AST segment is highly disordered in crystal structures while binding of the nucleotide drives the AST into an active and closed conformation. The C-terminal portion of the C-tail (residues 336–350) is anchored to the N-lobe in the active enzyme and is referred to as the N-lobe tether (NLT). At the end of the NLT is a hydrophobic motif (HM), and this motif (FXXF) is anchored to the αC-helix in the N-lobe (figure 5).

## 6. Kinases are dynamic switches

The EPKs are highly regulated enzymes that function as molecular switches, and PKA once again serves as a template to understand how the dynamic features that are a requirement for a ‘switch’ are implemented. Nuclear magnetic resonance studies of the PKA catalytic subunit, for example, have begun to explore the dynamic properties of the different conformational states of the catalytic subunit. They define the apoenzyme as a catalytically ‘uncommitted’ state where there are few backbone movements is the ms/μs time frame, the range that is important for catalysis [42,43]. Addition of the nucleotide creates a state that is ‘committed’ to catalysis where a network of correlated backbone motions throughout the protein are created. The peptide sequence containing the residue to be phosphorylated is also highly dynamic and is typically tethered in close proximity to the substrate through some distal site. These studies define a ‘molecular switch’ that is clearly distinct from metabolic enzymes that have evolved to be efficient catalysts and turn over large amounts of substrates. The EPKs, in general, are not efficient enzymes, and efficient catalysis is not a requirement for a switch.

## 7. Pre-steady-state versus steady-state kinetics

The EPKs are dynamic switches that are distinct from metabolic kinases such as hexokinases, which are molecular machines that turnover small molecules, often at or near rates limited only by diffusion and proportional to the concentrations of substrates. The other distinction between EPKs and metabolic kinases is the amount of substrates they encounter in the cell. There are usually millimolar concentrations of small molecules that get phosphorylated by other kinases, but EPKs and their protein substrates are present in submicromolar or even nanomolar concentrations. This substrate concentration difference between metabolic kinases and EPKs is one major reason why metabolic enzymes have high *K*_m_ values with efficient *k*_cat_ values while EPKs have low *K*_m_ values with relatively poor *k*_cat_ values. Because of these drastic concentration differences, EPKs have evolved efficient mechanisms such as scaffolding, small protein : protein interaction domains and peptide-binding pockets in order to increase the effective concentrations of EPK–substrate encounter complexes.

The basic tenet of mechanistic enzymology, carried over from metabolic enzymes, does not apply to the true physiology of EPKs. By this, we mean that steady-state kinetics and the apparent *K*_m_ and *k*_cat_ values are no longer relevant when concentrations of the enzyme and substrate are in the same range, sometimes with 1 : 1 stoichiometries. Thus, pre-steady-state kinetics with real numbers for the phosphoryl transfer step as well as *K*_d_ values are of critical importance to biophysically analyse EPKs. PKA was the first protein kinase to be comprehensively analysed by pre-steady-state kinetics [44], and this analysis revealed that the phosphotransfer step was significantly faster than the steady-state *k*_cat_ (500 versus 20 s^−1^). Such kinetics are consistent with the sort of rapid and transient reactions that have been optimized for specific and local phosphoryl transfers to proteins involved in a pathway, while minimizing non-specific phosphorylation of off-target proteins. Unfortunately, pre-steady-state kinetics is not a routine tool for investigating kinase mechanism, but we suggest that it should be routinely used to reveal new details of EPK mechanisms, and steady-state kinetics must be used as a preliminary guide.

Appreciating that efficient catalysis is not a feature that is essential for an EPK allows us to go back and look more carefully at the members of the kinome that were predicted to be pseudo kinases or ‘dead’ kinases. These kinases were predicted to be inactive because they lacked one or more of the conserved catalytic residues. However, a number of these have subsequently proved to be functional kinases. WNK (with no lysine (K)), for example, which lacked the lysine in β-strand 3 was found to have another spatially conserved basic residue that occupies the same space in the structure and serves the same functional role [45]. In the case of calcium/calmodulin-dependent serine protein kinase (CASK), which lacks the acidic residues that bind to the Mg^2+^ ions, it was found that the enzyme works on its specific substrate (neurexin) in the absence of Mg^2+^ [46]. Vaccinia-related kinase 3 (VRK3) is an example of a true pseudokinase where the space filled by the adenine ring in the C-spine is filled with aromatic side chains [47]. This kinase with its ‘fused’ spine is dead as a kinase but can still function as a scaffold. Kinase suppressor of Ras 1 (KSR) was also thought to be a ‘dead’ kinase that functioned only as a scaffold but it too is thought now to have kinase activity [48,49]. It is not essential for a kinase to be highly active, and in some cases, kinase activity is missed because the kinase is highly selective for a single substrate protein.

## 8. Regulation of protein kinase

Most of the EPKs are themselves phosphoproteins, and PKA serves as a template to understand how phosphorylation contributes to regulation. There are two sites of phosphorylation in the PKA catalytic subunit, Thr197 in the activation loop and Ser338 in the C-tail. Each contributes in unique ways to creating an active enzyme.

**Figure 6.**
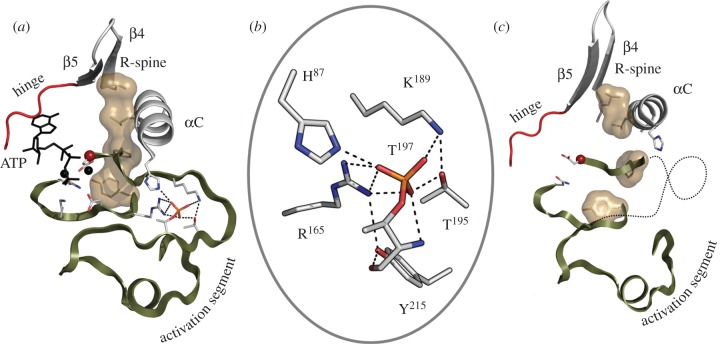
Phosphorylation of the activation segment drives the assembly of the R-spine. (*a*) The fully phosphorylated active PKA catalytic subunit shows how phosphorylation of Thr197 creates the contiguous R-spine that spans both lobes. (*b*) The activation loop phosphate interacts with five different subdomains. (*c*) In the absence of phosphorylation the R-spine is broken and the C-helix is pushed away from the active site.

Phosphorylation of the activation loop is essential for optimal PKA activity. As seen in figure 6, this phosphate, once in place, touches almost all of the motifs in the core. It allows us to appreciate how one moiety, even a single phosphate, can have such a profound effect. In the case of PKA, Thr197 can be autophosphorylated when it is expressed in *Escherichia coli*, so until recently, all of the PKA structures have been of the fully phosphorylated and active enzyme. By mutating the P-3 Arg in the activation loop, Arg194, we were able to purify a dephosphorylated enzyme that could then be phosphorylated *in vitro* by PDK1, which is a universal activating kinase for the AGC subfamily of EPKs. The C-subunit that lacks the phosphate on its activation loop is unstable and is also highly dynamic based on hydrogen deuterium exchange mapping by mass spectrometry (HDXMS), and in each case, the properties of the wild-type protein are restored by simply adding back the phosphate [50]. The catalytic properties of the enzyme are also significantly compromised. The *K*_m_ values for ATP and peptide are both increased. However, the most profound effect on catalysis is seen when one examines the pre-steady-state kinetics. PKA, such as a number of EPKs, shows an initial burst of activity that then levels off to give a steady-state rate of catalysis. The *k*_cat_ is regulated by the off-rate of MgADP. In the absence of the Thr197 phosphate, the pre-steady-state burst is virtually eliminated [51].

The crystal structure of the PKA C-subunit lacking its activation loop phosphate shows the profound effect that this phosphate has on the assembly of the active enzyme (figure 6). It demonstrates how the R-spine is broken and shows that the activation loop is now mostly disordered [51]. It also confirms the HDXMS results and illustrates how the various loops and segments of the C-subunit become more dynamic and in the case of the C-tail become disordered.

In almost every AGC kinase, there are two critical sites, in addition to the activation loop site, that are regulated by phosphorylation (figure 5). Between the AST and the NLT is a phosphorylation site that is referred to as the turn motif. In some cases, this is a *cis*-autophosphorylation site and occurs co-translationally as in the case of Akt [52], whereas in other cases, such as S6K, this site appears to be phosphorylated by a heterologous kinase [53]. With the exception of PKA and PKG, all other AGC kinases have an additional segment that is fused to the C-terminal HM, and a third phosphorylation site directly follows the HM. This HM site is highly regulated and is usually phosphorylated by the mammalian target of rapamycin (mTOR)-containing mTORC2 complex that is a multi-subunit complex protein kinase. In Akt, for example, this site is regulated by mTOR [52]. The HM site is turned over by a specific phosphatase called PHLPP [54]. As the AGC kinases are regulated by multiple phosphorylations that involve usually more than one heterologous kinase, it is of fundamental importance to study the order of the phosphorylation events and the effect that each phosphorylation has on the stability and activity of the kinase. Understanding the order and mechanism for regulation of the C-tail phosphorylation sites has been challenging, but it is essential to unravel this complexity if one is to appreciate how regulation is achieved at the molecular level.

In PKA, the C-tail site is most likely phosphorylated prior to the activation loop; however, once the two sites are phosphorylated they are very resistant to removal by phosphatases [55]. This is not true for other AGC kinases, and this allows the C-subunit to be packaged as part of a holoenzyme complex where activation is now completely dependent on the generation of cAMP. These complexes also recruit phosphatases, which is another reason why the PKA C-subunit itself needs to be resistant to phosphatases. In this way, the phosphatase is committed to the dephosphorylation of the protein substrate not to the dephosphorylation of the C-subunit. The only known physiological condition that makes the C-subunit susceptible to dephosphorylation is oxidation of Cys199, which happens only when the C-subunit is not inhibited by the R-subunit.

## 9. The protein kinase catalytic subunit is packaged as a holoenzyme

While we have learned much about the structure and function of PKA from studying the free C-subunit, in cells, it is assembled as a holoenzyme complex with inhibitory regulatory (R) subunits. Ultimately, it is the tetrameric holoenzyme complex that reflects the basal physiological state of the enzyme. In all mammals, there are four functionally non-redundant R-subunits (RIα, RIβ, RIIα, RIIβ), which all have a stable dimerization domain at the N-terminus. This is followed by a flexible linker that is classified as an intrinsically disordered region. Embedded within the linker is an inhibitor site that resembles a substrate and docks to the active site cleft in the holoenzyme. At the C-terminus are two CNB domains. In the absence of cAMP, two fully phosphorylated C-subunits bind to the R-subunit dimer rendering it inactive. The very stable helical dimerization domain is multi-functional. In addition to creating a stable dimer, it serves as a docking site for AKAPs. These are polyvalent scaffold proteins that are characterized by an amphipathic helix that binds with high affinity to the dimerization/docking (D/D) domain of the R-subunits [56]. Other signalling proteins such as phosphatases and phosphodiesterases are also anchored to AKAPs. The AKAP is then targeted in close proximity to specific substrates such as an ion channel, a transporter or a fusion protein on the mitochondria. In this way, the cell creates discrete ‘foci’ for cAMP signalling.

**Figure 7.**
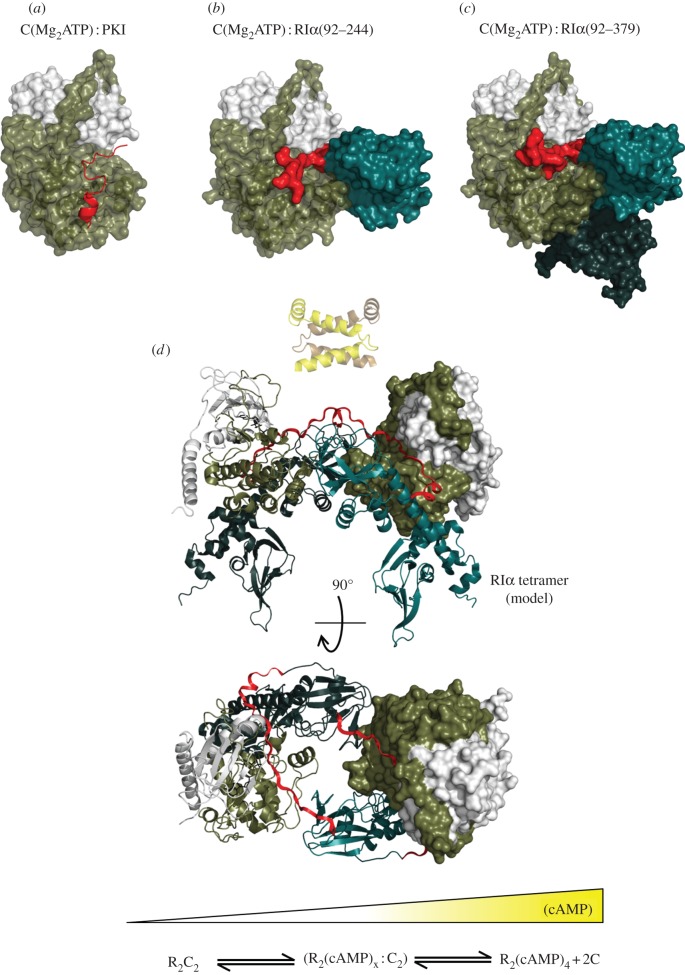
The catalytic subunit of PKA serves as a scaffold that interacts with multiple regulatory proteins. On the left (*a*) is a representation of the catalytic subunit bound to a peptide from the heat-stable protein kinase inhibitor (PKI) shown in red [22]. The middle (*b*) shows the catalytic subunit bound to a deletion mutant of RIα that contains a single nucleotide-binding domain (CNB-A) [57]. On the right (*c*) is the catalytic subunit bound to a deletion mutant of RIα that contains both single nucleotide-binding domains (CNB-A and CNB-B) [58]. The catalytic subunit is shown as a space-filling model with the residues from 1 to 126 in ivory and residues from 127 to 350 in tan. The R-subunits are also shown as space-filling models. The inhibitor site that docks to the catalytic site is shown in red. The CNB-A domain is in turquoise and the CNB-B domain is in dark teal. Panel (*d*) shows a model of the tetrameric RIα holoenzyme based on the crystal structure of a complex of a deletion mutant of the RIα subunit that contains an extended linker segment [59]. The dimerization domain shown in yellow is joined to the tetramer by a flexible linker. Rotation of the tetramer by 90°, minus the D/D domain shows how the N-linker of each heterodimer is docked onto the surface of the R-subunit CNB-A domain in the opposite heterodimer. This view also indicates the symmetry that is achieved in the tetramer. At the bottom is a gradient of cAMP, which regulates the activation and conformational state of the PKA holoenzyme.

Our understanding of PKA signalling has grown significantly as new structures were solved that included not only the free C-subunit but also complexes between the R- and C-subunits (figure 7). We did not understand, in molecular terms, how the C-subunit was actually regulated by the R-subunit until the structure of an R : C complex was solved [57]. This showed not only how the inhibitor site docked to the active site cleft of the C-subunit but also how the inhibition could be allosterically released by cAMP binding to the CNB domain. Two factors have further emphasized the importance of the complete full-length tetrameric holoenzymes. First, as discussed earlier, is that the C-subunit is unusual in its stability and in its resistance to phosphatases, which allows it to be packaged in a way that is sensitive exclusively to cAMP and not to the dynamic turnover of its activation loop phosphate. Second, is the solution of the structures of tetrameric holoenzyme complexes that allow us for the first time to appreciate the complex allosteric networks that are uniquely created in the tetramer [59]. It is only in the tetramer that one can appreciate the intricate symmetry of this enzyme. What is furthermore apparent is that each of the four tetrameric holoenzymes has a unique quaternary structure in spite of the highly conserved domain organization of each R-subunit and the very similar tertiary structure of each heterodimer. These differences, first predicted by small angle X-ray scattering studies [60,61], are now confirmed as different holoenzyme structures are revealed. The RIIβ holoenzyme, for example, is strikingly different from the RIα holoenzyme model [62]. It lends further credence to the idea that the PKA catalytic subunit is assembled as part of four functionally distinct holoenzyme complexes that are then localized to discrete foci in the cell where they are dedicated to the phosphorylation of a specific substrate or set of substrates that are co-localized in close proximity through scaffold and targeting proteins.

## 10. Conclusion

Clearly, one needs to now consider the entire system if one is to truly appreciate the complexity and beauty of how the eukaryotic cell has evolved to regulate its biological events by a highly dynamic process such as protein phosphorylation. One also needs to revisit the established paradigm that PKA signalling requires full dissociation of the R- and C-subunits. If activation occurs on a macromolecular complex that is anchored to a channel, for example, and co-localized with other signalling proteins such as phosphodiesterases, cyclases and/or phosphatases, it is not difficult to envision PKA signalling as an oscillatory process where full dissociation is not required. Such oscillatory circuits for PKA signalling have been reported recently for cAMP and calcium [63]. We know much about the structure and function of the PKA R- and C-subunits and are now learning about the unique isoform-specific features of the full-length tetrameric holoenzymes that are assembled in the absence of cAMP. As indicated in figure 7, PKA signalling almost certainly occurs somewhere in between these two endpoints in a time frame that probably does not allow for full dissociation and diffusion of the subunits. The initial discovery of protein phosphorylation in the middle of the last century thus continues to offer us new challenges and opportunities as we strive to understand how biological events are actually regulated in cells. It is a challenge that cannot be understood simply by studying one enzyme. Instead, one needs to understand how that molecule functions in the context of its environment and how that molecule is regulated by extracellular signals that communicate stress.
